# Supplementary material for: Active site specificity profiling datasets of matrix metalloproteinases (MMPs) 1, 2, 3, 7, 8, 9, 12, 13 and 14
Source: Data Brief. 2016 Feb 22;7:299–310. doi: 10.1016/j.dib.2016.02.036 (PMC4777984; doi:10.1016/j.dib.2016.02.036)
Supplement: Supplementary file 10 — Supplementary material [file mmc10.zip › WebPICS_hMMP14_G_1%/P1prime.html]

 

PICS results


|  |  |
| --- | --- |
| **P1prime\_C**  9 in 213 sites   4.2 %    effects > 10 perc. pnts.  (vice-versa in brackets)  P3\_C: 19.4 (29.1)   P3prime\_Q: 16.6 (12.5) |  |
| **P1prime\_F**  11 in 213 sites   5.2 %    effects > 10 perc. pnts.  (vice-versa in brackets)  P2\_R: 12.6 (11.5)   P2\_Y: 14.4 (19.8)   P2prime\_F: 12.1 (10.2)   P2prime\_R: 20.3 (14.8)   P3prime\_N: 21.7 (19.8) |  |
| **P1prime\_I**  36 in 213 sites   16.9 %    effects > 10 perc. pnts.  (vice-versa in brackets)  P2prime\_L: 19.2 (23.1)   P3prime\_A: 10.5 (15.1) |  |
| **P1prime\_M**  15 in 213 sites   7.0 %    effects > 10 perc. pnts.  (vice-versa in brackets)  P3\_H: 11.0 (33.0)   P2prime\_F: 13.9 (16.1)   P3prime\_T: 19.2 (18.0) |  |
| **P1prime\_V**  15 in 213 sites   7.0 %    effects > 10 perc. pnts.  (vice-versa in brackets)  P3\_P: 20.6 (11.5) |  |
| **P1prime\_Y**  7 in 213 sites   3.3 %    effects > 10 perc. pnts.  (vice-versa in brackets)  P3prime\_N: 23.0 (13.4) |  |
